# Restricting Online Sales and Reclassifying E-Cigarettes as Prescription-Only: A Public Health Policy Perspective

**DOI:** 10.7759/cureus.86222

**Published:** 2025-06-17

**Authors:** Taiwo O Aremu, Fredrick M Ogugua

**Affiliations:** 1 Division of Environmental Health Sciences, School of Public Health, University of Minnesota, Minneapolis, USA; 2 Department of Pediatrics, University of Minnesota School of Medicine, Minneapolis, USA; 3 Department of Pharmaceutical Care & Health Systems, College of Pharmacy, University of Minnesota, Minneapolis, USA; 4 Department of Cardiology, University of Illinois, Chicago, USA

**Keywords:** electronic cigarettes (e-cigarettes), electronic nicotine delivery systems (ends), health professional intervention, nicotine addition, online sales restriction, prescription-only regulation, public health policy, regulatory oversight, smoking cessation, youth vaping

## Abstract

The widespread use of electronic cigarettes, particularly among adolescents and young adults in the United States, has emerged as a critical public health challenge. Despite existing federal regulations, online access remains a significant channel for underage use, largely due to inconsistent enforcement of age verification. Although only about 2% of underage e-cigarette users report purchasing them online, more than three-quarters of online vendors allow checkout without verifying age, and test purchases frequently bypass identity checks, with nearly 70% resulting in delivery without age confirmation. E-cigarettes have been associated with numerous adverse health outcomes, including cardiovascular, respiratory, and neurological conditions. This editorial advocates for two urgent policy measures: a federal prohibition on the online sale of electronic nicotine delivery systems and the reclassification of these products as prescription-only. These steps would enhance regulatory oversight, limit youth access, and support evidence-based smoking cessation efforts under professional supervision. Implementing such reforms would strengthen public health protections, reduce healthcare costs, and promote long-term population well-being.

## Editorial

The rising use of electronic cigarettes, particularly among adolescents and young adults, presents an urgent public health concern in the United States. These products, known as electronic nicotine delivery systems (ENDS), were originally promoted as tools for harm reduction. However, their widespread availability, especially through internet-based platforms, has contributed to increasing non-therapeutic use, nicotine dependence, and the onset of chronic health conditions. A growing body of evidence now links e-cigarette use to cardiovascular disease, stroke, cancer, respiratory illness, and neurological harm, particularly in young individuals, highlighting substantial and well-documented health risks that justify stronger regulatory intervention [1,2].

Despite federal age restrictions and oversight by the Food and Drug Administration (FDA), e-cigarettes remain readily accessible online. While current laws prohibit sales to individuals under the age of 21 and require age verification during online transactions, enforcement in practice appears variable, allowing youth continued access through vendors with inadequate verification systems. Although the FDA has issued warning letters and imposed penalties in some instances, these actions tend to follow violations rather than prevent them. Compared to enhancing current enforcement measures, a complete federal prohibition on the online sale of e-cigarettes and similar products may offer a more definitive solution by removing this access point entirely. Such an approach would reduce underage use and address persistent weaknesses in the regulatory framework.

In addition to access restrictions, there is merit in reclassifying e-cigarettes as prescription-only products. Several countries have adopted this approach. For example, Australia implemented such a policy in 2021, requiring medical evaluation and oversight to obtain nicotine vaping products [3]. While long-term outcomes are still being assessed, the model highlights the importance of treating e-cigarettes as therapeutic tools rather than general consumer goods. Applying this framework in the United States would allow clinicians to determine whether e-cigarette use is appropriate within a structured smoking cessation plan. This would discourage recreational use, reduce inappropriate exposure, and promote medically supervised use.

Pharmacists, especially those practising in community settings, are commonly recognized as well positioned to support such a model. Their professional training in medication use and safety, combined with their accessibility, enables them to counsel patients, dispense products based on clinical need, and monitor ongoing use. Including pharmacists in the process would reinforce accountability and promote informed, patient-centered care.

The economic rationale for stronger regulation remains compelling. Smoking-attributable costs in the United States have been estimated at 289 to 332.5 billion dollars annually, based on data from 2009 to 2012 [4]. These costs include between 132.5 and 175.9 billion dollars in direct medical care for adults, approximately 151 billion dollars in lost productivity due to premature death, and 5.6 billion dollars in lost productivity from exposure to secondhand smoke [4]. While these figures reflect the broader impact of smoking, the growing association between e-cigarette use and conditions such as cardiovascular disease, stroke, and respiratory illness may introduce additional burdens to the healthcare system. Policies aimed at reducing nicotine exposure, including through e-cigarettes, can help alleviate future costs and support improved health outcomes over time.

Some may view these proposed measures as an infringement on individual choice or consumer freedom. Others may raise concerns about potential economic consequences, such as impacts on small retailers or industry profitability. These viewpoints merit consideration. However, they must be weighed against the public responsibility to reduce preventable harm. Nicotine addiction does not occur in isolation. It affects families, communities, and healthcare systems. Although the long-term health effects of e-cigarette aerosol are still being studied, precautionary principles suggest that limiting involuntary exposure may help protect vulnerable populations, particularly children and individuals with underlying health conditions. Public health policies often require carefully considered tradeoffs, and in this case, limiting access is justified by the need to protect population health and prevent addiction-related harm.

Implementation will not be without obstacles. Opposition may arise from industry stakeholders, civil liberty advocates, and some consumers. Nonetheless, the data are clear. The continued growth of e-cigarette use among youth puts public health gains at risk. Flavored products remain a major driver of this trend, with recent surveys showing that nearly nine out of ten youth who use e-cigarettes prefer flavored options [5]. Without timely and decisive policy action, we may be enabling a new wave of nicotine addiction with consequences that will echo for decades.

Health professionals, public health advocates, policymakers, regulatory agencies, and public health organizations must work collaboratively to update national policies in alignment with emerging evidence and international standards. The United States, long regarded as a leader in science and public health innovation, now has an opportunity to reaffirm that role. Ending internet sales of e-cigarettes and requiring a prescription for access would represent a meaningful step toward reducing nicotine dependence, protecting young individuals, and promoting a healthier future for the country.
